# Comparison of Mesh and Barbed Suture for Laparoscopic Nephrosplenic Space Ablation in Horses

**DOI:** 10.3390/ani11041096

**Published:** 2021-04-12

**Authors:** Rodolfo Gialletti, Sara Nannarone, Marco Gandini, Anna Cerullo, Alice Bertoletti, Nicola Scilimati, Gessica Giusto

**Affiliations:** 1 Department of Veterinary Sciences, University of Turin, 10095 Grugliasco, Italy; rodolfo.gialletti@unipg.it (R.G.); sara.nannarone@unipg.it (S.N.); alicebertoletti1@gmail.com (A.B.); nicola.scilimati@yahoo.it (N.S.); 2 Department of Veterinary Medicine, University of Perugia, 06126 Perugia, Italy; anna.cerullo@unito.it (A.C.); gessica.giusto@unito.it (G.G.)

**Keywords:** colic, nephrosplenic entrapment, nephrosplenic space ablation, laparoscopy, barbed suture, prosthetic mesh

## Abstract

**Simple Summary:**

The entrapment of the left large colon in the nephrosplenic space is a common cause of colic in horses but not in miniature horses and pony breeds. Different techniques have been proposed to prevent this problem, such as colopexy, large colon resection or nephrosplenic space (NSS) ablation. The most recent methods involve closing the NSS with a minimally invasive technique with the horse standing. Multiple techniques have been described for laparoscopic NSS ablation, among which are ablation with a barbed suture and ablation with prosthetic mesh. Although these two methods have been proposed as valid and effective, no direct comparison has yet been made between them. In this study, the two techniques are compared in terms of duration of surgery, short- and long-term complications, and costs.

**Abstract:**

Nephrosplenic space (NSS) ablation has been demonstrated to be an effective technique for prevention of left dorsal displacement of the large colon and multiple laparoscopic techniques, among which ablation with mesh or with a barbed suture, have been proposed. Our objective was to compare two laparoscopic techniques for closure of the NSS in twenty-eight horses diagnosed with nephrosplenic entrapment. Medical records of horses that had laparoscopic NSS ablation in two referral centers between 2017–2019 were retrieved. Duration of surgery, complications, and short- and long-term follow-up information were collected and compared. Costs were also calculated and compared. All horses met the inclusion criteria: 9 had NSS ablation with a mesh implant (group M), 19 with barbed suture material (group B). One horse in group B had recurrent colic after discharge. At control laparoscopy after 5 months, the NSS resulted in still not being ablated because of a failure of the suture. In group M, three horses had recurrent colic. One was successfully treated medically, one died of unknown causes and the third required a second laparoscopic suturing at 3–6 months because of failure of the mesh implant. The mean time of surgery and costs were lower in group B compared to group M. The barbed suture technique was faster, more cost-effective and had a lower complication rate than the mesh implant.

## 1. Introduction

Left dorsal displacement of the left large colon (LDDLC) is one of the displacement of the large colon in horses but not in miniature horses and pony breeds [1,2,3]. Although the causes of the entrapment of the left colon in the nephrosplenic space (NSS) has not been completely elucidated as an initial or recurrent disease, some theories have been proposed that can be referred to because of the dysfunction of the colon motility and/or the excessive accumulation of gas [4,5,6]. The “V” shape and the depth of the NSS could be relevant predisposing factors [7,8]. A great variability in the recurrence rate of LDDLC is described and ranges between 3.2% and 20.7% [6,8]. Surgical interventions that can prevent the recurrence of this displacement are colopexy, partial resection of the colon and closure of the NSS [8,9]. The reported surgical techniques include ventral laparotomy or flank laparotomy in general anesthesia or with the horse standing, and laparoscopy [5]. The most recent methods involve closing the NSS with a minimally invasive technique with the horse standing [1,6,10]. Several techniques have been described for laparoscopic ablation of the NSS such as ablation with barbed suture [1] and ablation with a prosthetic mesh [3,10]. Although these techniques require somewhat advanced surgical skills, the use of specific material such as the barbed suture or the application of the prosthetic mesh can make the procedure easier.

Although these two methods have been proposed as valid and effective, no direct comparison has yet been made between them to verify if one technique is superior to the other. Our hypothesis is that closure of the NSS with barbed sutures is faster and is likely to cause fewer complications than closure with mesh.

Thus, the objective of the study is to compare two different laparoscopic techniques for closing the NSS, in terms of surgery time, postoperative complications and costs.

## 2. Materials and Methods

Medical records of horses diagnosed with LDDLC at the Large Animal Teaching Hospitals of the University of Turin and University of Perugia between January 2017 and December 2019 were analyzed. Horses enrolled in the study had a diagnosis of LDDLC, some resolved spontaneously during hospitalization and others underwent exploratory laparotomy. Criteria for proposing a laparoscopic closure of the NSS were horses diagnosed with LDDLC, recurrent episodes treated medically or with a single episode of LDDLC that required celiotomy. The owner signed an informed consent before any procedure. Before surgery, horses received a complete physical examination (temperature, heart rate, respiratory rate, mucous membrane color and capillary refill time). The NSS was evaluated by palpation per rectum, which is the mainstay of diagnosis of NSS because the left colon in most cases could be palpated into the nephrosplenic space. The transabdominal ultrasound examination should be used in conjunction with rectal palpation findings. In horses with NSS, the presence of gas-filled colon dorsal to the spleen almost always precludes imaging the kidney [11]. Water was freely available, but food was withheld for 24 h before surgery to reduce the amount of ingesta in the large colon, to minimize the risk of iatrogenic damage to viscera during cannula placement and to optimize laparoscopic visualization. All horses received pre-operative antibiotics (procaine penicillin G 22,000 IU/kg IM and gentamicin sulfate 6.6 mg/kg IV), anti-inflammatory (flunixin meglumine 1.1 mg/kg EV) and tetanus prophylaxis if not vaccinated (5000 IU IM). A 14-gauge IV catheter was placed into the jugular vein and horses were sedated with detomidine (8.4 µg/kg IV) and placed in stocks for the standing procedure. Sedation was maintained via continuous rate infusion of detomidine (0.5–0.1 µg/kg/min IV) with a syringe pump. The left paralumbar fossa was clipped and the surgical field was aseptically prepared and draped. The three portal sites were locally infiltrated with 15–20 mL 2% lidocaine hydrochloride in the skin, fascia and muscles. The first portal was created with a 10-cm-long, 10-mm-diameter blunt trocar-cannula system (Versaport V2, Covidien Italia S.p.A., Segrate, Milano, Italy) caudally to the margin of the 18th rib, in the cranial third of an imaginary line passing through the left tuber coxae [12]. Subsequently, a 0° angle, 33-cm-long laparoscope (Striker, San Jose, CA, USA) was inserted. The abdomen was first explored but not insufflated. Two additional portals were created under direct vision. The second one was created in the 17th intercostal space at the level of the ventral aspect of the tuber coxae with a 10-cm-long, 12-mm-diameter trocar-cannula system (Versaport V2, Covidien Italia S.p.A., Segrate, Milano, Italy) and a 10-cm-long, 5-mm-diameter trocar-cannula system (Versaport V2, Covidien Italia S.p.A., Segrate, Milano, Italy) was introduced in the third portal, which was created 5 cm ventral to the first one. For surgery, the laparoscope was then moved to the second port. At the end of surgery, the trocars were removed and the anatomical planes reconstructed: the muscle fascia was closed with a simple continuous suture of resorbable USP 1, the skin was closed with a simple interrupted suture of USP 0 nylon thread. Surgery time was recorded from skin incision to placement of the last skin suture.

### 2.1. Surgical Technique with Barbed Suture

**Figure 1:**
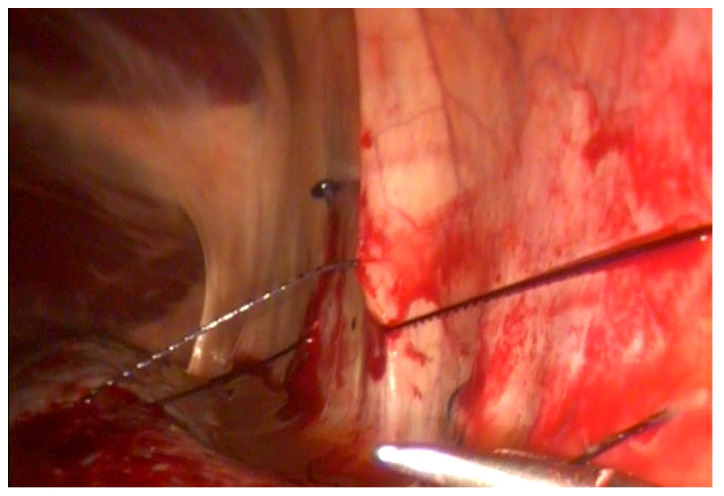
NSS ablation with barbed suture (Filbloc^®^). Note the button at the end of the thread that facilitates application of the first bite.

To obliterate the NSS, a continuous suture was used with a 30-mm-long, one-half-circle taper needle attached to loop-ended, USP 0 absorbable polydioxanone knotless suture material (V-Loc 90, Covidien Italia S.p.A., Segrate, Milano, Italy, as previously described or with a 26-mm-long, one-half circle taper needle attached to USP 2 absorbable polydioxanone self-retaining barbed suture with a PDO button in the end (Filbloc^®^Assut Europe S.p.a., Magliano dei Marsi, L’Aquila, Italy). The closure of the NSS occurs through the apposition of the suture between the perirenal fascia and the dorso-medial aspect of the fibrous capsule of the spleen in a cranio-caudal orientation [6]. The first step of the suture is performed in the most cranial part of the ligament, with the dorso-ventral direction of the needle. The needle passes at the same height on the spleen capsule with ventro-dorsal direction. The barbed suture, after the first passage, is self-retained. The simple continuous suture is continued at intervals of about 1.5 cm to obliterate the space (Figure 1). To end the suture, two additional backwards passages were performed. When the suture is finished, laparoscopic scissors are used to cut the thread.

### 2.2. Surgical Technique with Mesh Application

**Figure 2:**
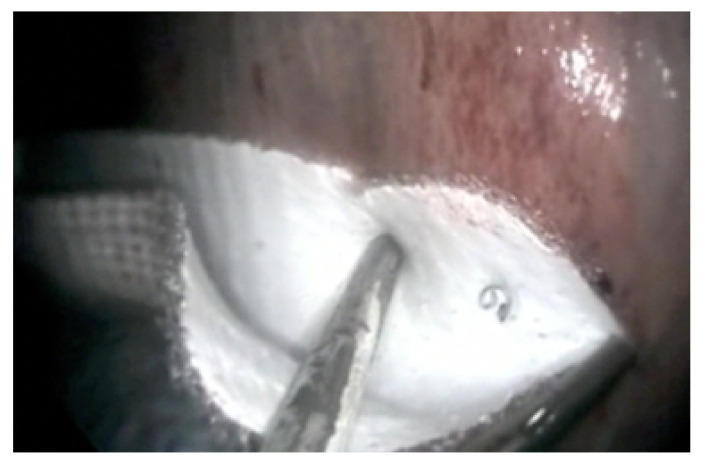
Nephrosplenic Space (NSS) ablation with mesh apposition.

The required mesh size was estimated by using a 5-mm probe with 1 cm demarcations inserted through the second instrument portal. The width of the mesh was 1–2 cm greater than the measured distance from the spleen to the perirenal fascia to allow a slight concavity in the mesh once in place to prevent excessive tension at the site of insertion. A 7.6 cm × 15.2 cm Bard^®^ Composix Mesh (Becton Dickinson Italia S.p.A, Milano, Italy) monofilament knitted of polypropylene and expanded polytetrafluoroethylene (ePTFE) mesh was rolled into a 1 cm cylinder and its shape retained with 2 encircling sutures (3-0 polyglactin 910), each tied with a square knot. Laparoscopic Endo-Babcock™ forceps (Medtronic Italia S.p.A., Milano, Italy) were used to introduce the mesh cylinder through the first instrument portal into the abdominal cavity. Once the mesh was introduced into the abdominal cavity, with the help of two Endo-Babcock™ forceps, the net was properly positioned ensuring that the polypropylene side was in the medial portion and therefore in contact with perirenal band and spleen, while the ePTFE layer was in the side portion towards the bowels. The longer edges of the mesh were placed along the back-medial margin of the spleen, the ligament and the perirenal capsule to create a link between these anatomical portions. The mesh was fixed by inserting the Covidien ProTack™ 5 mm Fixation Device (Covidien Italia S.p.A., Segrate, Milano, Italy) in the third trocar. The assistant surgeon kept the mesh in tension to ensure that the relevant bodies were properly adhered. Titanium helical coils were applied to fix the mesh into the soft tissues (Figure 2). An average of 20 coils, spaced about 1.5 cm apart, were used depending on the need for the case based on the size of the space. In one case, to facilitate mesh application, after insertion of a 18G 3.5” spinal needle as a guide to avoid thoracic penetration, a fourth portal was created at the 16th intercostal space (just dorsal to the second portal), which allowed the entrance of another Laparoscopic Endo-Babcock™ forceps, and consequently a better grip on the mesh.

### 2.3. Postoperative Management

After recovery from standing sedation, horses were placed back into their stable and were allowed to access food after about 3–4 h. Postoperative therapy included the administration of gentamicin (6.6 mg/kg, SID, IV), procaine benzylpenicillin (20,000 IU/kg, BID IM) and flunixin-meglumine (1.1 mg/kg, SID IV). Horses were monitored for postoperative complications, defined as those occurring from the end of surgery to hospital discharge. Horses were discharged after 3–7 days with instructions for 2 weeks of stall rest followed by 2 weeks of 15–20 min of hand walking twice a day. Since a non-absorbable suture was used for closing the skin at the portals, removal at 2 weeks was suggested.

Follow-up was obtained by telephone interview of owners at 12–24 months from discharge. Mean time of surgery and cost of procedures were recorded and compared between groups.

### 2.4. Statistical Analysis

The study power and sample size were calculated with a web-based calculator with an alpha level set at 0.05 and 90% power using the mean of surgery time. Weight and age of horses were compared to assess the omogeneicity of groups. Surgical time and complication rate were compared between groups. Normality of data was assessed with the Shapiro–Wilk test. For normally distributed variables, parametric tests were used, while non-parametric tests were used in the case of not normally distributed data. Fischer’s exact test was used to compare odds ratio for complications. Significance was considered for *p*-Values < 0.05. Statistical analysis was performed using commercial software (Prism 8.0, GraphPad, San Diego, CA, USA).

## 3. Results

Twenty-eight horses underwent NSS obliteration and met the criteria for the study. According to the surgical technique used to ablate the NSS, two groups were defined. Group B included horses in which NSS was obliterated with barbed suture (*n* = 19), and group M included horses in which NSS was obliterated with mesh (*n* = 9). The median age of the horses in group B was 12 (range 2–21 years) and 9 years (range 5–14) in group M (*p* = 0.183, Mann–Whitney). The median weight was 480 kg (385–680) in group B and 570 (400–600) in group M (*P* = 0.785, Mann–Whitney). There were 15 geldings, 8 mares, and 5 intact males. The breed distribution included Italian warmblood (*n* = 10), KWPN (*n* = 5), Thoroughbred (*n* = 4), French warmblood (*n* = 3), Belgian warmblood (*n* = 2), QH (*n* = 1), Paint (*n* = 1), Arabian (*n* = 1), and Frisian (*n* = 1). All horses had the diagnosis of nephrosplenic entrapment confirmed either by rectal and ultrasonographic examination (cases treated medically) or at laparotomy.

The mean surgical time was significantly shorter in group B than in group M (62.00 ± 17.10 and 78.44 ± 14.55 min, respectively, *p* = 0.017, unpaired *t*-test). All 28 horses survived to hospital discharge; however, short-term complications were recorded and limited to mild edema of the laparoscopic portals, without any differences among the two groups. No other complications related to the procedure were reported before discharge. Long-term follow-up, ranging from 24–48 months, was available for all the horses. Twenty-six horses (93%) were alive at the time of long-term follow-up. One out of 19 horses in group B and 3 out of 9 horses in group M developed complications (odds ratio 9, *p* = 0.08), while 8 horses underwent second-look laparoscopy (Table 1).

**Figure 3:**
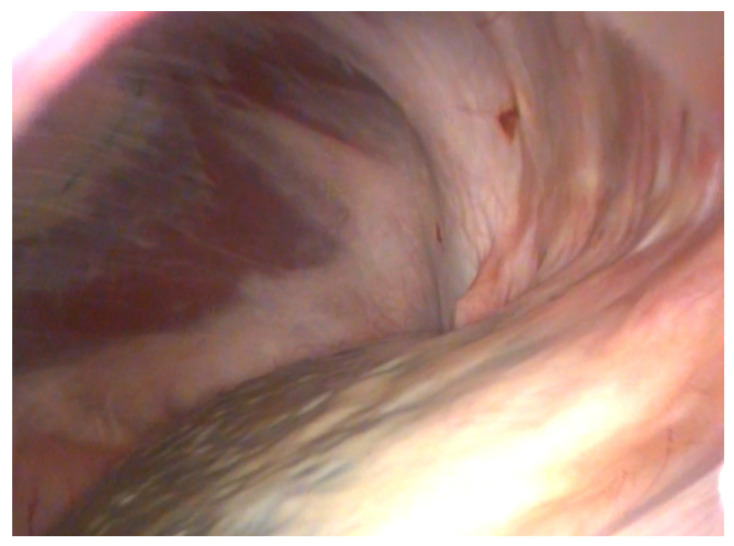
Second-look laparoscopy of an NSS space ablation with barbed suture.

Four horses out of the 19 in group B underwent second-look laparoscopy (one for postoperative complications, the remaining three for other laparoscopic procedures) and in three horses neither adhesions nor other complications were observed and the NSS appeared fully ablated (Figure 3). One horse had recurrent colic after discharge. At control laparoscopy after 5 months, the NSS resulted in still not being ablated because of failure of the suture. The nephrosplenic space was further closed with barbed suture and at 6 months of follow-up the owner reported complete resolution of recurrent colic.

In group M, four horses out of 9 underwent control laparoscopy (three for postoperative complications, one for other laparoscopic procedures): one horse did not show adhesions nor other complications and the NSS appeared fully ablated; two horses had the NSS fully ablated but adhesions were observed between coils and mesocolon and the large colon.

One horse had recurrent colic and laparoscopic control at 3–6 months revealed failure of the mesh to fully ablate the NSS, which required a second surgery. After re-suturing the horse did not show any further sign of colic. One horse in group M had one episode of large colon impaction within 2 years of laparoscopic surgery that resolved with medical treatment and another one died 24 months after first laparoscopy for unknown causes and necropsy was not performed.

Twenty-four horses (85.7%) returned to their previous activity and two horses out of four were retired from sporting activity for reasons other than gastrointestinal concerns.

The difference in costs was compared by evaluating only the specific equipment used for the two techniques and not the entire cost of surgery. The cost was estimated in 20 and 480 euro in group B and M, respectively.

## 4. Discussion

Laparoscopy is a minimally invasive technique, which can be performed with the horse either recumbent or standing, with a consequent difference in overall costs and recovery time.

We described the successful closure of the nephrosplenic space in 24/28 horses with a history of recurrent LDDLC using an uncomplicated laparoscopic surgical technique and no evidence of recurrent LDDLC over 2 years. Two techniques have been described and compared: in group B, the ablation of the NSS with barbed suture was used and in group M a mesh implant was carried out. In group B there were fewer complications then in group M. The barbed suture technique was cheaper than the mesh implant and allowed a faster surgical time then the mesh implant.

In this study, several breeds were included; however, there was no difference in the mean weight between groups. In our clinical experience, horses heavier than 550 kg have a deeper abdomen, resulting in an increased risk of an inability to suture the entire length of the nephrosplenic ligament and in a more uncomfortable working position for the surgeon. This increases the risk of not performing the surgical procedure accurately.

An advantage of the suturing technique is that the barbs provide many anchoring points on the tissues, allowing for a more even distribution of the tissue holding forces over the entire length of the suture [13]. This suture should therefore be less likely to induce complications such as failure of the suture knot, the main cause of which is localized excessive strain. In addition, the barbed suture increases the adhesion surface between the tissues, favoring the healing process [1].

Ablation of the NSS with a laparoscopic barbed suture appeared to be slightly more difficult because it required more steps in order to complete the suture. However, the duration of surgery was shorter in group B than in group M. Furthermore, it is important to note that even if the application of the mesh with coils is hypothetically easier than suturing, if the mesh or coils were applied under tension, a failure of the mesh fixation would occur, with a consequent failure to ablate the NSS.

One horse in group B had the NSS still not ablated at laparoscopic control. The failure of the suture was likely caused by improper placement of one or more sutures during the procedure or by improper placement of a trocar that did not allow adequate access to the tissues.

One horse in group M had recurrent colic and required suturing with barbed suture at 3–6 months due to failure of the mesh implant to completely ablate the NSS. In this horse, the mesh had been placed with excessive tension on the soft tissues and some coils were placed improperly causing the mesh to move.

Indication of NSS ablation reported to reduce the incidence of recurrent colic as well as to prevent incarceration in the NSS [2]. Some authors reported that the closure of the NSS by laparoscopy reduces the incidence of recurrent colic [2,14]. Colic episodes recorded in our study after the NSS ablation included other types of large colon displacements and some mild colic treated medically.

In accordance with Albanese et al. [1] and Munoz et al. [15], CO_2_ insufflation was not used in any of the procedures in this study. It has already been reported that the dorsal position of the anatomical area of interest allows visual manipulation even without abdominal distension, especially if the horse is fasted for 24–36 h before surgery. A reported and time-consuming technical complication in group B was the involuntary tangle of the suture thread during the procedure [1]. In our case, a difficult step when using V-Loc 180^h^ barbed suture material was the insertion of the needle through the small, welded loop. To overcome this issue, we created a large suture loop prior to introduce the suture into the abdomen, which enabled to secure the first bite and eliminated this difficult step [6]. Furthermore, the use of a thread with a PDO button (Filbloc^®i^) at the end eliminated the previous difficulty and did not involve any inconvenience in performing the suture.

In horses where suture was used to close the NSS, no intra-abdominal adhesions were observed compared to those where a mesh was implanted. In our study, adhesions were found between the coils and the mesocolon in one horse, and between the mesh and the large colon in another one. However, adhesion formation has been already described in horses where mesh and coils have been used to close the NSS [16].

Polypropylene nets have been reported for years to increase the risk of adhesions also in human surgery [17], while polytetrafluoroethylene (ePTFE) nets are rarely associated with adhesion formation both in horses and in rabbit models [16,18]. For this reason, we used the ePTFE mesh. This material is unlikely to induce adhesions; however, we hypothesized that some tacks could have damaged the surface of the mesh during placement. Such damage might have exposed a portion of polypropylene which then favored the formation of adhesions with the mesocolon and the large colon.

Furthermore, some authors have argued that a great advantage was to perform a technique without excessive tension on the tissues [10]. Continuous barbed suture provides more anchor points and therefore a more even distribution of traction forces, increasing the surface of adhesion and aiding in the healing process [19].

Nephrosplenic ablation with the mesh occurs when the fibrous tissue is included around the mesh, incorporating it into the surrounding tissue; therefore, it takes time for the space to close. In contrast, suturing techniques depend on suture strength and tissue integrity to maintain immediate space closure after surgery is completed.

Some authors [5,20] have postulated that a large and deep NSS in some horses is the major predisposing factor for nephrosplenic entrapment, but objective measurements of NSS in horses with and without colon entrapment have not been described.

The technique used in group B had more advantages than the mesh implantation used in group M such as a relatively easier placement, which has led to a faster procedure, a lower incidence of postoperative adhesion formation, and a lower cost.

The main limitations of this study were a different sample of the two groups, and we included laparoscopy performed in two hospitals with different surgeons, although the centers worked with the same methods. In both techniques, there was a learning curve and with practice, the surgical time decreased. We believe that the practice has decreased the difficulty of performing the correct placement of the laparoscopic ports, the correct placement of the instruments and a better manipulation of the surgical instruments in all surgeons.

Both methods have been effective in ablating the NSS, but barbed suture reduces the surgery time, is cheaper than the mesh implantation, and reduces the risk of adhesions, post-operative colic, and the risk of ablation failure [2,12,21].

## 5. Conclusions

The results of our study show that barbed suture is an effective surgical technique for closing the NSS and provides surgeons with an alternative to mesh implant and obliteration with a non-barbed suture. The decision on which technique should be chosen should rely upon the availability of equipment, the surgeon’s level of comfort with suturing techniques, and the cost of the procedures.

## Figures and Tables

**Table 1:** Details of horses that underwent second-look laparoscopy.

-	Group B (*n* = 19)		Group M (*n* = 9)	
Total	4	-	4	-
Complications	1	-	3	-
Re-suture	-	1	-	0
Adhesions	-	0	-	2
Failure of mesh	-	0	-	1
No complications	3		1	-
Orchiectomy	-	2	-	0
Uterine imbrication	-	0	-	1

## Data Availability

The data presented in this study are available on request from the corresponding author.
